# Frustrated Magnetism in Triangular Lattice TlYbS_2_ Crystals Grown via Molten Flux

**DOI:** 10.3389/fchem.2020.00127

**Published:** 2020-02-26

**Authors:** Timothy Ferreira, Jie Xing, Liurukara D. Sanjeewa, Athena S. Sefat

**Affiliations:** Oak Ridge National Laboratory, Materials Science and Technology Division, Oak Ridge, TN, United States

**Keywords:** flux growth, single crystal, frustration, quantum materials, magnetism, triangular lattice

## Abstract

The triangular lattice compound TlYbS_2_ was prepared as large single crystals via a molten flux growth technique using sodium chloride. Anisotropic magnetic susceptibility measurements down to 0.4 K indicate a complete absence of long-range magnetic order. Despite this lack of long-range order, short-range antiferromagnetic interactions are evidenced through broad transitions, suggesting frustrated behavior. Variable magnetic field measurements reveal metamagnetic behavior at temperatures ≤2 K. Complex low temperature field-tunable magnetic behavior, in addition to no observable long-range order down to 0.4 K, suggest that TlYbS_2_ is a frustrated magnet and a possible quantum spin liquid candidate.

## Introduction

The variety of basic and applied properties arising from geometrically frustrated magnets continue to motivate the study of structures with two-dimensionally layered triangular lattices prone to antiferromagnetic interactions (Chubokov and Golosov, 1991; Lee et al., 2006; Shen et al., 2016; Li et al., 2017a; Zhu et al., 2018; Bordelon et al., 2019; Ranjith et al., 2019). Such triangular lattices restrict the number of available spin degrees of freedom, resulting in quantum fluctuations that can produce degenerate ground states (Savary and Balents, 2016). Frustrated antiferromagnets with degenerate ground states have garnered significant interest for their potential as quantum spin liquid (QSL) candidates, a state characterized by dynamic entangled spins, exhibiting no long-range magnetic order, even at 0 K (Anderson, 1973; Balents, 2010; Hu et al., 2015; Starykh, 2015; Yamamoto et al., 2015; Dun et al., 2016; Savary and Balents, 2016; Shen et al., 2016; Xu et al., 2016; Li et al., 2017b; Paddison et al., 2017; Baenitz et al., 2018). While QSL candidates with 3*d* ions exhibit weak spin-orbit coupling (SOC) (Lee and Lee, 2005; Helton et al., 2007; Yoshida et al., 2009; Yamashita et al., 2010; Zhou et al., 2011; Shirata et al., 2012), the presence of stronger SOC in 5*d*/4*f* ions, on par with the energy scale of crystal electric field effects and the coulomb interaction *U*, further enhances the frustration via entangled spin and orbital degrees of freedom, and has thus shifted the search for new QSL materials to contain these heavier lanthanides (*Ln*) (Okamoto et al., 2007; Curnoe, 2008; Gardner et al., 2010; Onoda and Tanaka, 2010; Applegate et al., 2012; Hu et al., 2015; Li et al., 2016; Lu et al., 2017; Laconis et al., 2018; Liu et al., 2018).

Recently, Yb(III) containing compounds such as NaYbO_2_ (Bordelon et al., 2019; Ranjith et al., 2019), NaYbS_2_ (Baenitz et al., 2018), and YbMgGaO_4_ (Li et al., 2015, 2017a,b; Xu et al., 2016; Paddison et al., 2017) have been presented as QSL candidates, all crystallizing in a layered triangular lattice of trigonal space group *R*$\bar{3}$*m*. Due to the odd number of 4*f* electrons and strong SOC, these materials behave as effective spin *J*_eff_ = ½. A similar family of 4*f*-containing delafossites, of the general formula A(I)*Ln*(III)*Ch*(II) [A = Na, Rb, K; *Ch* = O, S, Se, Te] (Liu et al., 2018), has also been proposed as a promising candidate. Delafossites are often free from crystallographic site-mixing, unlike the more commonly studied YbMgGaO_4_ that can mimic QSL behavior by eliminating long-range order through disorder (Li et al., 2017b; Zhu et al., 2017). Additionally, the modular nature of delafossite structures allows for the possibility of differences in crystal structure as a function of the ratio of ionic radii; this has been reported to result in changes in triangular lattice layer stacking, such as ABAB stacking in the hexagonal *P*6_3_/*mmc* or ABCABC layer stacking in the trigonal *R*$\bar{3}$*m*. Reports of possible inter-layer interactions on the highly sensitive magnetic ground state of such systems makes the delafossite structure advantageous to study, as such subtle interactions can be probed as a function of selective ion control (Yamamoto et al., 2015).

Despite the structural modularity and promise of QSL candidacy in such delafossite structures, the limited availability of large single crystals to study electronic and magnetic anisotropy serves as motivation for this work. Herein we discuss the flux crystal growth, structure determination, and magnetic property measurements of TlYbS_2_, which crystallizes in the hexagonal space group *P*6_3_/*mmc*. This study allows for proper structure elucidation of TlYbS_2_ single crystals, contrary to the results of polycrystalline powders (Duczmal and Pawlak, 1994), in addition to reporting novel anisotropic magnetization results that were inaccessible on polycrystalline samples (Duczmal and Pawlak, 1994).

## Experimental

### Synthesis

The TlYbS_2_ compound was synthesized using a two-step method comprised of (1) producing the powder form via traditional solid-state synthesis, followed by (2) crystallizing the precursor powder using molten flux growth via sodium chloride. Solid pieces of Yb metal (REacton, 99.99%), S (Puratronic, 99.9995%), and Tl (REacton, 99.99%) were all stored in a glove box. Sodium chloride (Alfa Aesar, 99.999%) was dried in an oven overnight at 300°C and stored in a desiccator prior to use.

**Figure 1:**
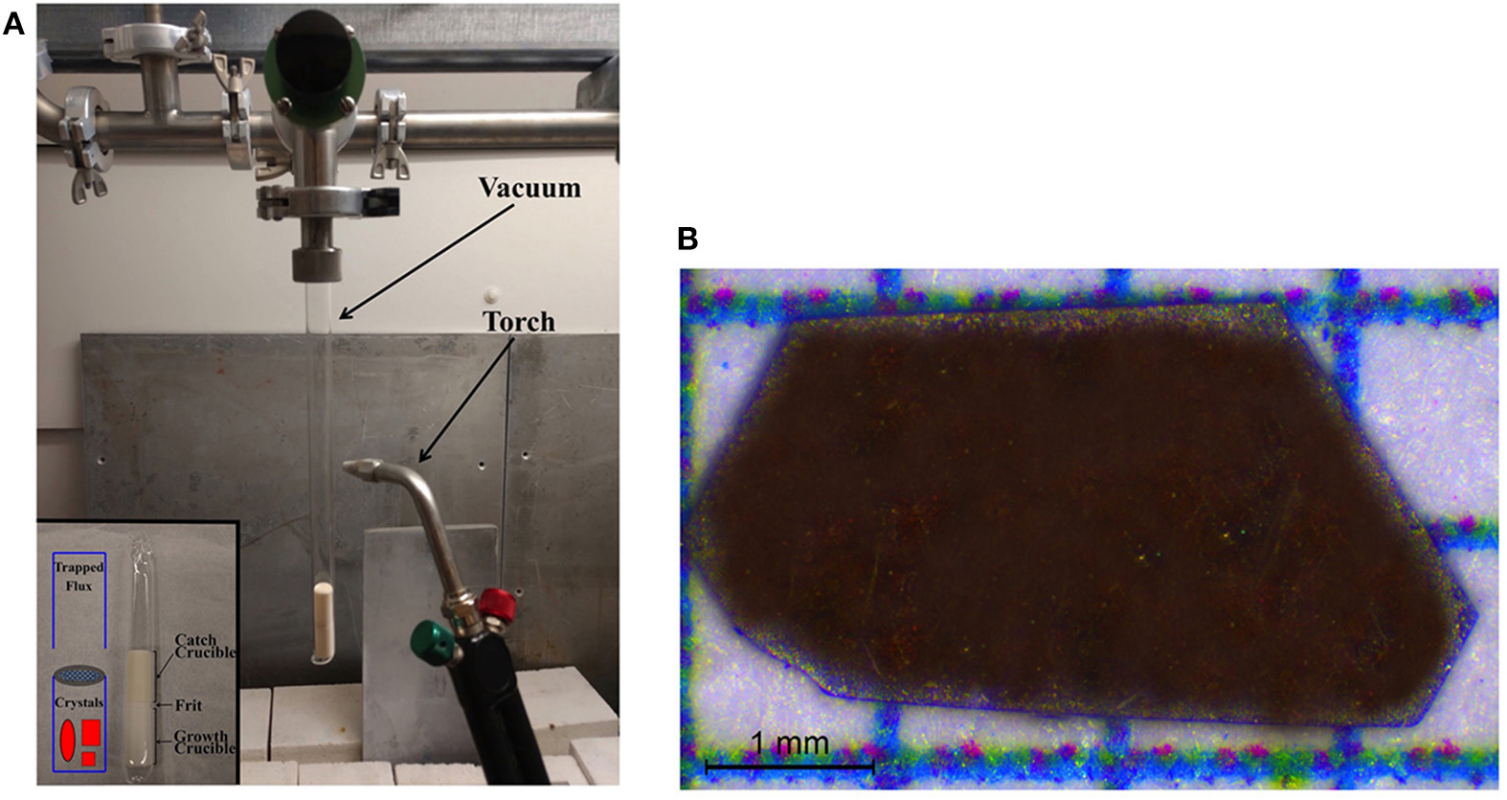
**(A)** Experimental setup consists of an alumina crucible housing the starting materials with a loose-fitting frit and a second inverted alumina crucible, shown in the inset, both held within a sealed evacuated quartz tube. **(B)** Optical image of a typical crystal of TlYbS_2_, showing its red color. The *c* axis is out of the plane of the crystal.

For the solid-state synthesis, 2.0 mmol of Tl and Yb were added to 4.0 mmol of S in an alumina crucible with a loose-fitting alumina frit and a second, inverted, alumina crucible on top. This second crucible was used to assist in catching any trace amounts of volatilized Tl or S. This setup of alumina crucibles was loaded and sealed inside an evacuated silica tube, with a small amount of quartz wool at the bottom to prevent cracking due to differences in thermal expansion (Figure 1A). The sealed silica tube was heated to 300°C at a rate of 10°C/h, dwelled for 24 h, ramped to 800°C at a rate of 10°C/h, dwelled at 72 h, and then the reaction was allowed to cool by shutting off the furnace.

**Figure 2:**
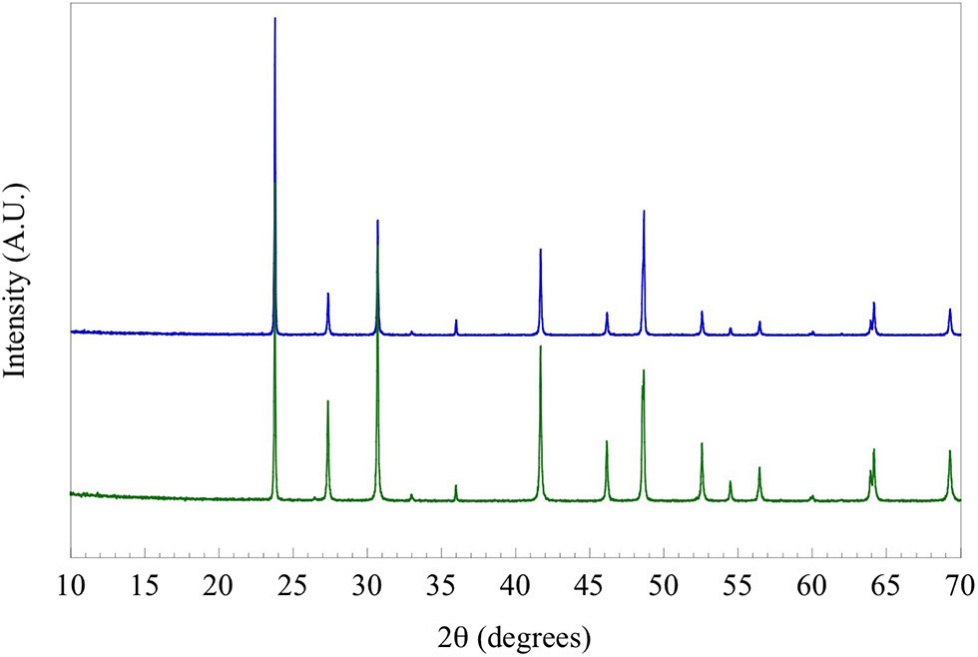
Powder X-ray diffraction pattern of solid-state produced polycrystalline TlYbS_2_, shown in blue, overlaid with a diffraction pattern of ground single crystals of TlYbS_2_, shown in green.

Single crystals were produced by loading 0.87 mmol of polycrystalline TlYbS_2_ and a ten-fold excess (by mass) of NaCl (40.47 mmol) into sealed evacuated silica tubing. The reaction was heated to 850°C at a rate of 60°C/h, dwelled at 504 h (i.e. 3 weeks), and then cooled by shutting off the furnace. The resulting red crystals were mechanically separated from the remaining TlYbS_2_ powder and vacuum filtered using ethanol to remove any surface impurities (Figure 1B). The crystallographic *c*-axis is out of the plane of the paper. The purity of the polycrystalline powder (first step) and resulting crystals (second step) were both determined by powder X-ray diffraction (PXRD) using a PANalytical X'Pert Pro MPD diffractometer with Cu Kα1 radiation (λ = 1.5418 Å), shown in Figure 2.

### Structure

The structure determination of the single crystals of TlYbS_2_ was performed on a Bruker Quest D8 single crystal X-ray diffractometer (SXRD). Single crystals were first sonicated in acetone to remove any surface impurities. The data were collected at room temperature utilizing a Mo Kα radiation (λ = 0.71073 Å). The crystal diffraction images were collected using Φ and ω-scans. The diffractometer was equipped with an Incoatec IμS source using the APEX III software suite for data setup, collection, and processing (Bruker, 2015). The structure was resolved using intrinsic phasing and full-matrix least square methods with refinement on *F*2 using the SHELXTL software suite (Sheldrick, 2008). All atoms were first refined with isotropic thermal displacement parameters and then refined anisotropically. Crystallographic information can be found in Tables 1–3.

**Table 1:** Crystallographic data for TlYbS_2_ determined by single crystal X-ray diffraction.

**Empirical formula**	**TlYbS_**2**_**
Formula weight (g/mol)	441.53
*T*, K	273
Crystal habitat	Red plates
Crystal dimensions, mm	0.060 ×0.020 × 0.020
Crystal system	Hexagonal
Space group	*P*6_3_/*mmc* (No. 194)
*a*, Å	3.9454(8)
*c*, Å	15.277(7)
Volume, Å^3^	205.94(12)
*Z*	2
*D* (calc), g/cm^3^)	7.120
μ (Mo Kα), mm^−1^	62.409
*F*(000)	366
*T*max, *T*min	0.2092–1.0000
θ range	2.67–30.63
Reflections collected	1,816
Data/restraints/parameters	102/0/9
Final *R* [*I*> 2σ(*I*)] *R*_1_, *R*_w2_	0.0366/0.0797
Final *R* (all data) *R*_1_, *R*_w2_	0.0402/0.0825
GoF	1.069
Largest diff. peak/hole, e/Å^3^	1.831/−1.820

**Table 2:** Atomic coordinates and equivalent isotropic displacement parameters (Å^2^) for TlYbS_2_.

**Atom**	***Wyckoff***	***x***	***y***	***z***	***Ueq* (Å^**2**^)**
Tl	2*c*	0.33333	0.66667	0.25000	0.0471 (10)
Yb	2*b*	0	0	0	0.0268 (8)
S	*4f*	0.33333	0.66667	0.5965 (6)	0.0278 (19)

**Table 3:** Bond distances (Å) and angles (°) for TlYbS_2_.

	**Bond distances (Å)**		**Bond Angles (^**°**^)**
Yb–S (6x)	2.713 (5)	Yb–S–Yb	93.3 (2)
Yb–Yb	3.9454 (8)	Yb–Yb–Yb	60.0

Energy dispersive spectroscopy (EDS) was performed using a Hitachi S-3400 scanning electron microscope equipped with an OXFORD EDX microprobe to confirm the elemental composition in the single crystal sample. The presence of Tl, Yb and S were verified, and the absence of extraneous elements such as Na and Cl were confirmed. EDS data can be found in Table 4.

**Table 4:** Energy dispersive spectroscopy data.

**Element**	**Atomic %**
Tl	25.99
Yb	25.10
S	48.91

### Magnetic Susceptibility

Physical properties were measured using the Quantum Design Superconducting quantum interference device (SQUID) Magnetic Properties Measurement System (MPMS). Two thin plates of crystals were stacked with a total mass of 0.36 mg using vacuum grease, for each measurement above 2 K. The magnetization measurements made below 2 K were measured using the Quantum Design iHe3 option on four stacked single crystals (0.5 mg total) using vacuum grease. The crystals were aligned in two separate orientations: one set of measurements aligned the crystals such that the applied magnetic field was perpendicular to the *c*-axis, and second set where the applied field was parallel with *c*. The thermometer calibration was done in zero field when the applied field was parallel with c.

## Discussion

### Synthesis

The scarcity of known single crystal growth methods for lanthanide containing delafossites, particularly those employing the use of salt flux, has made the optimization and characterization of large single crystals of the titled composition challenging (Stowe, 1997). Fortunately, there is significant literature evidence demonstrating that the use of molten flux as a growth medium is a robust method, potentially capable of crystallizing nearly every element combination on the periodic table (Bugaris and zur Loye, 2012). The selection of an alkali halide flux was guided in part by recent reports of similar fluxes crystallizing compositions containing lanthanides and/or chalcogenides (Klepov and zur Loye, 2018; Tsujimoto et al., 2018; Usman et al., 2019a,b). Additionally, amongst the few delafossite-type structures reported as single crystals, synthesis typically involves the use of reactive alkali fluxes, such that the alkali metal in the flux incorporates into the final product, such as the use of KCl for KErSe_2_ (Xing et al., 2019a) or NaCl for NaYbS_2_ (Baenitz et al., 2018). For producing TlYbS_2_ crystals, the use of TlCl as a flux medium was ruled out due to the low solubility prohibiting ease of crystal separation upon completion of the reaction. We tried CaCl_2_ as a flux, and although single crystals were produced, they were of poor quality. Attempts to improve the quality of the crystals by introducing cooling rates also failed: a variety of polycrystalline powders and no crystals were present, indicating TlYbS_2_ may be a metastable kinetic phase that is “trapped” via quenching. Ultimately, the use of NaCl as a flux, in addition to quenching the reaction upon the completion of the prolonged dwelling period, resulted in high quality, large (1 mm+) single crystals that were suitable for structural and anisotropic magnetization studies.

### Structure

**Figure 3:**
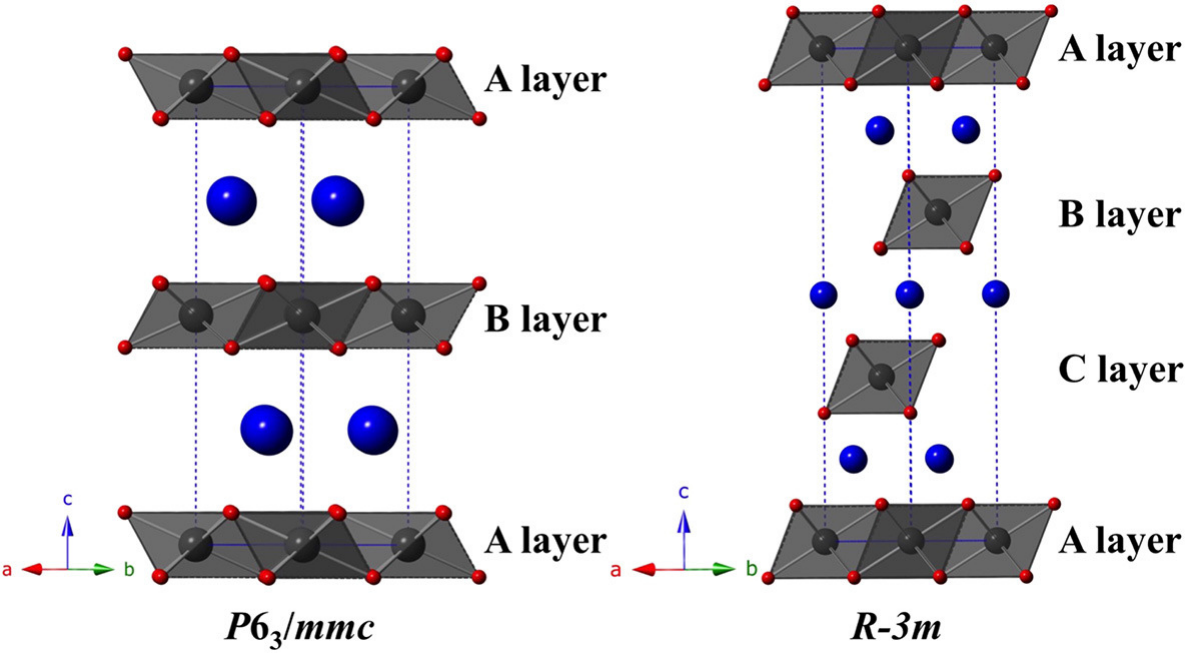
Polyhedral representation of the delafossite structure, with the hexagonal *P6*_3_*/mmc* variant (shown left) and the trigonal *R*$\bar{3}$*m* variant (shown right). The hexagonal structure is built of three distinct triangular layers of edge-shared YbS_6_ octahedra (Yb shown in gray, S in red) that stack in an ABAB order, meanwhile the trigonal structure is built from four distinct triangular layers of edge-shared YbS_6_ octahedra that stack in an ABCABC sequence. Non-magnetic TI atoms are shown as blue spheres.

The compound TlYbS_2_ was first reported as a polycrystalline powder crystallizing in the trigonal space group *R*$\bar{3}$*m*, commonly referred to as the α-NaFeO_2_ structure, with lattice parameters *a* = 3.935 Å and *c* = 22.47 Å (Duczmal and Pawlak, 1994). This two dimensional layered structure is built from two distinct triangular lattice layers that alternate along the *c* axis. The first layer is built from a network of edge-shared octahedral YbS_6_ units that adopts an ABCABC stacking pattern (Figure 3). A second non-magnetic triangular lattice layer of edge-shared octahedral TlS_6_ units resides between these layers. As a result of the availability of single crystals of the titled composition, we thoroughly investigated the nuclear structure. Careful analysis revealed that the grown single crystals of TlYbS_2_ adopt the hexagonal *P*6_3_/*mmc* β-RbScO_2_ structure type. In this hexagonal structure, Tl (Wyckoff 2*c*), Yb (2*b*), and S (4*f*) occupy the special positions with site symmetries of −6*m*2, −3*m*, and 3*m*, respectively. This is in contrast to the previously reported trigonal *R*$\bar{3}$*m* structure for polycrystalline powders of TlYbS_2_ where Tl (3*b*) and Yb (3*a*) are in the −3*m* position, and S (6*c*) is in the 3*m* special position. The primary difference between the two structures is best understood by the number and stacking sequence of the triangular lattice layers. In the hexagonal β-RbScO_2_ structure, one unit cell is built from three YbS_6_ layers and two TlS_6_ layers, whereas the trigonal α-NaFeO_2_ structure unit cell is built from four YbS_6_ layers and three TlS_6_ layers. Additionally, the hexagonal β-RbScO_2_ structure adopts a higher symmetry ABAB triangular lattice layer stacking, in contrast to the ABCABC layer stacking in the trigonal α-NaFeO_2_ type structure. This result is consistent with the trend observed for smaller A-site ions in the delafossite structure crystallizing in the trigonal system, such as NaYbS_2_ (Baenitz et al., 2018) and larger A-site ions crystallizing in the hexagonal system, such as CsYbSe_2_ (Xing et al., 2019b). Comparison of powder X-ray diffraction patterns of solid-state produced polycrystalline powder, and single crystals grown via molten flux, of TlYbS_2_ overlay well (Figure 2) with slight differences in peak intensity and crystallinity. Although polymorphism between the polycrystals and single crystals should not be completely ruled out, slight differences in peak intensity may result from preferred orientation in such a highly anisotropic nuclear structure.

**Figure 4:**
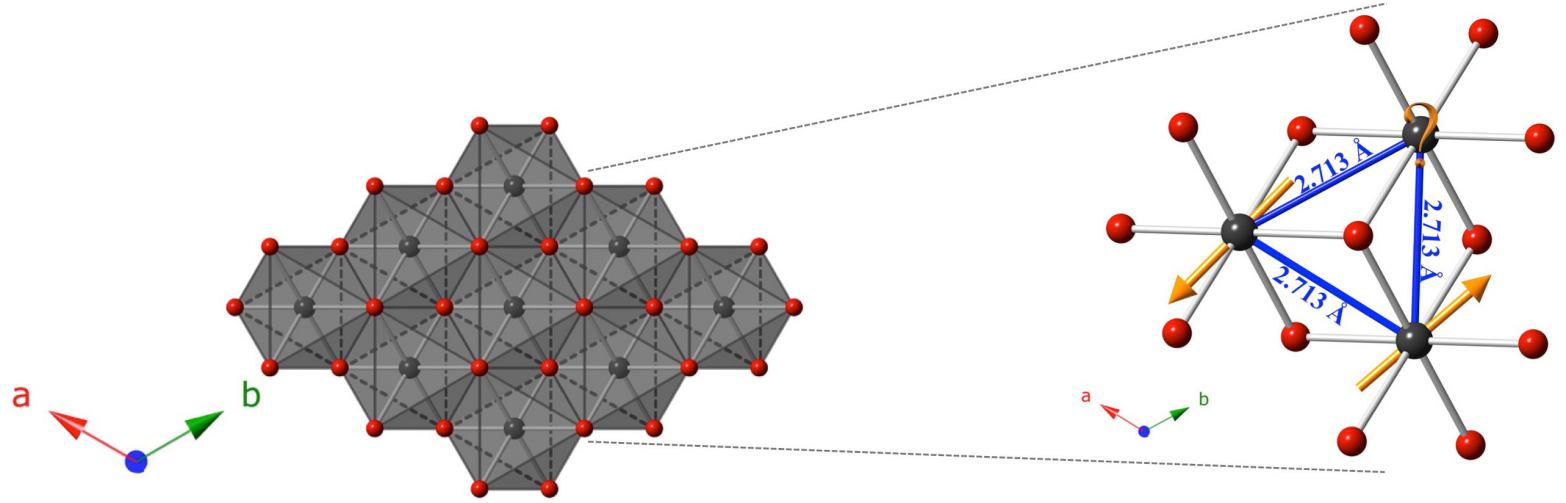
Polyhedral representation of the edge-shared octahedral network of YbS6 extending in the *ab* plane for the composition TlYbS_2_. The planar triangular lattice is an ideal host for geometric frustration for an antiparallel alignment of spins, as shown in the zoomed-in area. Yb atoms are shown in gray, and S atoms are shown in red.

The primary interest in studying this structure type is the triangular lattice that extends along the *ab* plane, serving as an ideal host for geometric frustration (Figure 4). The idealized hexagonal network of Yb atoms does not allow for a purely antiparallel configuration of spins, leading to enhanced quantum fluctuations that are of interest to study. Careful attention was taken to select Tl as the A site in the delafossite structure, since it is larger than Yb, both to prevent crystallographic site-mixing, and to maximize the interlayer distances, thereby minimizing inter-layer interactions via the mediating cation. The reactive-flux nature of NaCl in the growth of similar delafossite structures also guided the selection of the large cation Tl, in hopes that Na would be too small to occupy the same site. Additionally, a non-magnetic A site was selected to further simplify study of any resulting magnetic properties.

### Magnetic Susceptibility

**Figure 5:**
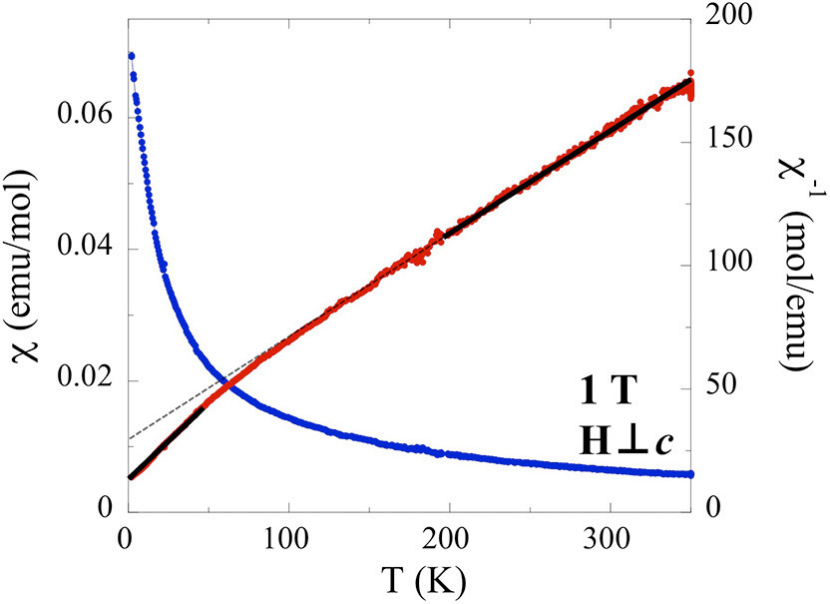
Temperature dependence of the magnetic susceptibility for single crystals of TlYbS_2_, oriented H⊥*c* under an applied field of 1 T. Field cooled data is shown in blue and inverse magnetic susceptibility data shown in red. Solid black lines denote the high/low temperature fits of the inverse susceptibility data.

The temperature dependence of the magnetization perpendicular to the applied field (in the *ab* plane) at 1 T indicates a broad deviation from Curie-Weiss behavior at 50 K (Figure 5). A Curie-Weiss fit of the high temperature section (200–350 K) of the inverse susceptibility taken at 1 T indicates a Weiss constant of θ_CW_ = −101.3 K and an effective moment of 4.57 μ_B_, in good agreement with the expected *J* = 7/2 (4.54 μ_B_) moment (Hashimoto et al., 2003). The large negative Weiss temperature indicates strong antiferromagnetic interactions. A fit of the low temperature (2–50 K) taken at 1 T indicates a smaller Weiss constant of θ_CW_ = −19.5 K and an effective moment of 3.41 μ_B_. To better understand the significant difference between high temperature moment [*J* = 7/2 (4.54 μ_B_)] and low temperature moment (3.41 μ_B_, unknown *J*), a literature search for analogous results was conducted. Similar measurements (also taken at low temperature and field) on structurally related (α-NaFeO_2_-type) single crystals of NaYbS_2_ made in-plane (*ab* plane) report comparable values of θ_CW_ = −13.5 K and μ_eff_ = 3.2 μ_B_ (Baenitz et al., 2018). The slight deviation in value between the two structures may originate from differences between Na and Tl ionic radii, [1.02 and 1.50 Å, respectively] causing the structural shift from *R*$\bar{3}$*m* (closer layers) to *P*6_3_/*mmc* (greater inter-layer distances). As a result of the larger non-magnetic cation (Tl) further separating the triangular lattice layers, TlYbS_2_ more closely resembles a pure two-dimensional structure, compared to the Na analog. The report on NaYbS_2_ describes the effective magnetic moment (μ_eff_ = 3.2 μ_B_) to originate from strong SOC and *ab* plane anisotropy, treating the studied material as a *J*_eff_ = ½ system according to the two-dimensional spin ½ triangular lattice Heisenberg antiferromagnet model (Anderson, 1973; Huse and Elser, 1988). This Heisenberg model has been used to understand previously reported isostructural frustrated systems in which all magnetic ions are 120° from one another, which is the case for NaYbS_2_, and thus is reasonable as a starting hypothesis for a possible magnetic model to explain the behavior of TlYbS_2_ (Liu et al., 2018). To assess the *J*_eff_ = ½ magnetic model, describing the proposed low temperature behavior for the Yb atoms in NaYbS_2_, Baenitz et al. conducted electron-spin-resonance (ESR) measurements on single crystals and found the *g*-factor to be highly anisotropic along the two crystallographic directions (*g*_*ab*_ = 3.19; *g*_c_ = 0.57), an expected consequence of the triangular lattice layer (Baenitz et al., 2018). Utilizing the experimentally determined *g*_*ab*_-factor, the *J*_eff_ = ½ magnetic model hypothesis, and considering Equation (1), an expected moment (μ_eff_) was calculated and found to be close to their experimentally observed moment of 3.2 μ_B_, suggesting that NaYbS_2_ behaves as *J*_eff_ = ½ triangular lattice Heisenberg antiferromagnet.

(1) $$\begin{array}{r} \mu_{eff}=g\sqrt{J\;\left(J+1\right)} \end{array}$$

**Figure 6:**
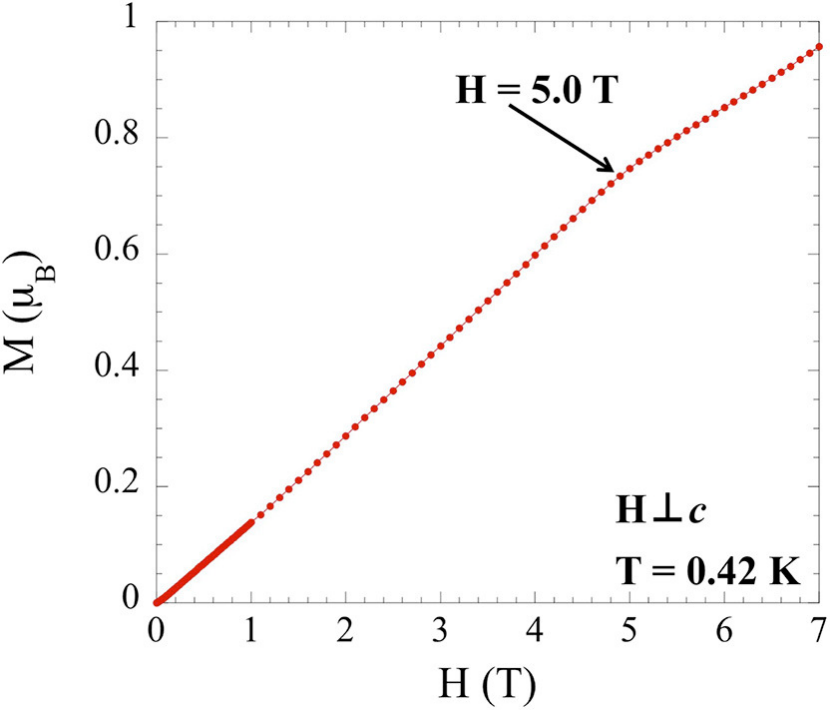
Field dependence of the magnetization for single crystals of TlYbS_2_ oriented H⊥c over a range of 0 to 7 T at 0.42 K.

Based on the success of the analysis used for NaYbS_2_, a similar method was employed for TlYbS_2_ to understand the low temperature magnetic moment. Within the triangular lattice Heisenberg AFM model, the magnetization should plateau at ~1/3 the expected saturation magnetization, according to Equation (2). In this equation, the saturation magnetization

(2) $$\begin{array}{r} m_{s\;}=J\;\cdot g \end{array}$$

(*m*_*s*_) is equal to the product of the *g*-factor and the total angular momentum (*J*). By analyzing TlYbS_2_ in the same way NaYbS_2_ was treated using Equation (1), an anisotropic *g*-factor of *g*_*ab*_ = 3.94 for TlYbS_2_ is obtained. This can be used in Equation (2) to obtain a saturation magnetization saturation (1.97 μ_B_). Taking the ratio of the moment (0.74 μ_B_) corresponding to the inflection point observed in field dependent magnetization measurements taken at 0.42 K (Figure 6) with the *m*_*s*_ discussed above yields a value of 0.37, in good agreement with the expected 0.33. It should be noted that in the absence of a sufficiently strong magnetic field, a full plateau is not observed, but the inflection point is the onset of such a plateau. Considering the triangular lattice orientation of the Yb atoms in the *ab* plane, as shown in Figure 4, and the good agreement of 0.37 to the expected 0.33 as per the triangular lattice Heisenberg AFM model, it is reasonable to propose a magnetic model in which TlYbS_2_ behaves as a *J*_eff_ = ½ system in the low temperature (below 50 K) regime. This finding is further supported by previous reports of subtle changes in slope for inverse magnetic susceptibility plots, such as that observed in Figure 5, as being attributed to gradual transitions toward an isolated Kramers doublet ground state (Ranjith et al., 2019).

**Figure 7:**
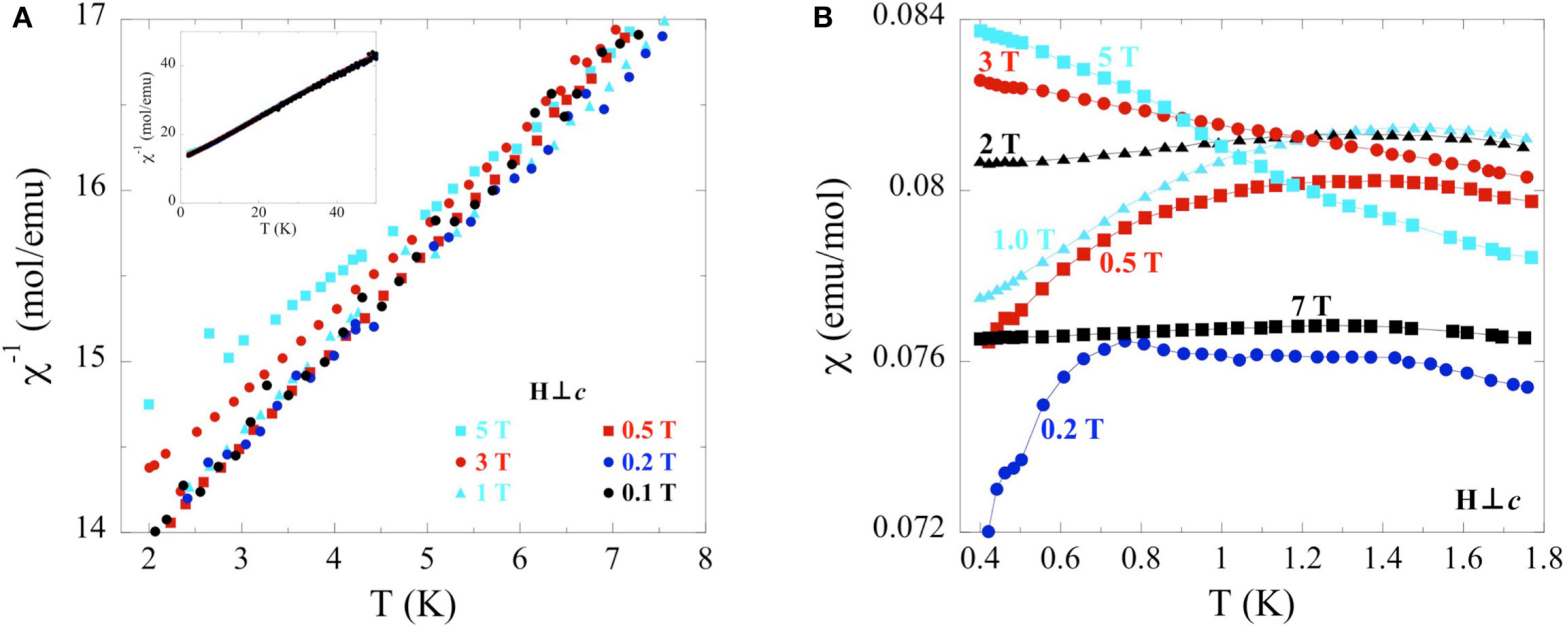
**(A)** Temperature dependence (2–8 K) of inverse magnetic susceptibility for single crystals of TlYbS_2_ oriented H⊥*c* under applied fields ranging from 0.1 to 5 T. A wider temperature range (2–50 K) of the sample plot is shown in the inset. **(B)** Temperature dependence (0.4–1.8 K) of susceptibility under applied fields from 0.2 to 7 T.

The broad deviation from Curie-Weiss behavior at 50 K was investigated further by measuring inverse temperature dependent susceptibility perpendicular to the field at low temperatures (2–50 K) at fields from 0.1 to 5 T (Figure 7A). A clear deviation from linearity can be seen in the 3 T data at 4 K, with a more pronounced deviation at the same temperature for the 5 T data. Supplementary temperature dependent susceptibility measurements (Figure 7B) taken at low temperatures (0.4–2 K) and variable fields (0.2–7 T) reveal a gradual inversion of slope, with the 7 T data appearing as a nearly flat line. The absence of saturation in the field dependence magnetization measurements made in Figure 6 at 7 T suggests the nearly linear susceptibility at 7 T in Figure 7 does not correspond to true saturation.

**Figure 8:**
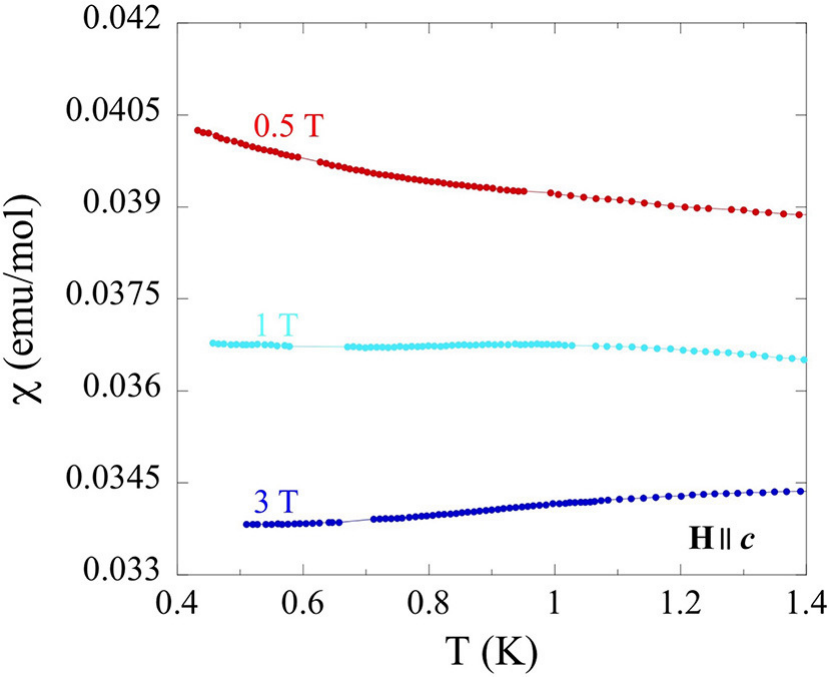
Temperature dependence (0.4–1.4 K) of magnetic susceptibility for single crystals of TlYbS_2_ oriented H||*c* under applied fields from 0.5 to 3 T.

Similar, albeit more subtle, features were observed in low temperature (0.4–1.4 K) susceptibility measurements (Figure 8) at variable fields (0.5–3 T), along the *c* axis. The presence of such very subtle transitions and an even weaker magnetic susceptibility perpendicular to *c* are unsurprising, as magnetic interactions are expected to reside primarily within the triangular lattice *ab* plane. The large size of non-magnetic Tl atoms that separate the triangular lattices reasonably limit inter-layer magnetic interaction pathways. Although a complete description of this complex behavior is not possible with the current data, similar behavior has been recently reported in Os_0.55_Cl_2_ (McGuire et al., 2019) and MErSe_2_ (M = Na, K) (Xing et al., 2019a). In both reports, no long-range ordering and a spin-liquid-like behavior is observed in triangular lattice frameworks similar to TlYbS_2_, suggesting that it may also be a candidate for this exotic behavior.

## Conclusions

In summary, for the first time large single crystals of TlYbS_2_ were prepared from a molten flux growth technique, and a reinvestigation of the nuclear structure was conducted. The single crystal structure was determined to crystallize in the hexagonal *P*6_3_/*mmc* β-RbScO_2_ type structure, in contrast to the previously reported trigonal *R*$\bar{3}$*m* α-NaFeO_2_ structure for polycrystalline samples. Anisotropic measurements along the *ab* plane and along *c* ranging from 0.42–350 K and 0.1–7 T displayed clear short-range magnetism and metamagnetic behavior. The complete absence of long-range order and consistency of TlYbS_2_ magnetic behavior to the two-dimensional triangular lattice Heisenberg antiferromagnet model indicates its possible candidacy as a quantum spin liquid.

## Data Availability Statement

The datasets generated for this study can be found in the Cambridge Crystallographic Data Centre (https://www.ccdc.cam.ac.uk/structures/) under the identifier 1965470. Alternatively, email data_request@ccdc.cam.ac.uk, or by contact The Cambridge Crystallographic Data Centre, 12 Union Road, Cambridge CB21EZ, UK; fax +44 1223 336033.

## Author Contributions

TF was responsible for making the materials in polycrystalline and single crystal forms; additionally, he drafted this manuscript. JX was responsible for the magnetic measurements taken, and LS was responsible for the structural characterization. AS oversaw the experiments and helped in finalizing this manuscript.

### Conflict of Interest

The authors declare that the research was conducted in the absence of any commercial or financial relationships that could be construed as a potential conflict of interest.
